# Determining the state of guidance on pediatric biobanking for researchers, HRECS, and families: Regulatory mapping of international guidance

**DOI:** 10.1007/s00431-024-05469-8

**Published:** 2024-03-13

**Authors:** Sinead Prince, Shih-Ning Then, Kerry-Ann O’Grady

**Affiliations:** 1 Australian Centre for Health Law Research, Faculty of Business and Law, Queensland University of Technology, Brisbane, QLD Australia; 2 Centre for Healthcare Transformation, Australian Centre for Health Services Innovation, Faculty of Health, Queensland University of Technology, Brisbane, QLD Australia

**Keywords:** Pediatric, Biobank, Regulation, Guidance, Law, Ethics

## Abstract

Biobanking—the storage of human biological samples, including tissue, blood, urine, and genetic data—raises many ethical, legal, and social issues, including confidentiality and privacy. Pediatric biobanking is more complicated, with difficulties arising because children lack capacity to consent and acquire this capacity upon maturity when the research is still ongoing. Yet given the limited availability of pediatric samples, the translational nature of biobanking presents a unique opportunity to share samples and produce clinically necessary information about pediatric development and diseases. Guidance on navigating these legal and ethical difficulties is needed for those involved in pediatric biobanking—including researchers, participants, and families, and those involved in biobank governance. This paper seeks to map the current regulatory framework governing pediatric biobanking to determine what guidance is currently offered. Regulatory mapping of current international and national guidelines on pediatric biobanking addressing the ethical, legal, and social nuances of pediatric biobanking was undertaken. This paper finds that international guidelines around biobanking are mostly for adults, and even when pediatric-specific, documents are non-binding, inconsistent, or only limited guidance is offered on a range of important issues specific to pediatric biobanks.

*Conclusion*: This paper shows a need for consistent, comprehensive, and clear regulation on pediatric biobanking so that research can more quickly, efficiently, and ethically be translated to useful information and treatment in pediatric care.

**Table:** 

**What is Known:** *• Pediatric biobanking presents new opportunities to conduct valuable translational research to benefit pediatric populations. However, the storage of pediatric biological samples raises many ethical, legal and social issues—in part because child participants may be considered to lack capacity to consent but can acquire this capacity upon maturity when the research is still ongoing. Pediatric biobanks must grapple with issues of consent, confidentiality and privacy, and long-term participation regarding child participants.*	
**What is New:** *• Regulatory guidance on these ethical, legal, and social issues is needed for researchers, participants, and families and those involved in biobank governance. This paper identifies nationally specific and international guidance on biobanking and summarizes the guidance provided in relation to these pediatric specific issues. It finds that most guidance is non-binding and inconsistent between guidance documents and may offer only limited guidance to stakeholders. A need for consistent, comprehensive, and clear regulation on pediatric biobanking is needed at an international level to enable research.*

**What is Known:**

*• Pediatric biobanking presents new opportunities to conduct valuable translational research to benefit pediatric populations. However, the storage of pediatric biological samples raises many ethical, legal and social issues—in part because child participants may be considered to lack capacity to consent but can acquire this capacity upon maturity when the research is still ongoing. Pediatric biobanks must grapple with issues of consent, confidentiality and privacy, and long-term participation regarding child participants.*

**What is New:**

*• Regulatory guidance on these ethical, legal, and social issues is needed for researchers, participants, and families and those involved in biobank governance. This paper identifies nationally specific and international guidance on biobanking and summarizes the guidance provided in relation to these pediatric specific issues. It finds that most guidance is non-binding and inconsistent between guidance documents and may offer only limited guidance to stakeholders. A need for consistent, comprehensive, and clear regulation on pediatric biobanking is needed at an international level to enable research.*

## Introduction

Biobanks enable the high-quality, long-term, mass storage of human biological samples for translational, interdisciplinary research. Samples include tissue, blood, urine, and genetic data derived from such sources that come from clinical research participants or as by-products with consent from patients receiving treatment. While the exact numbers of biobanks that exist globally are unknown, interest in the establishment and ethical governance of such entities grows, as evidenced by increased weight given to relevant accreditation, training, and scholarly discourse [1, 2]. While only a proportion of existing biobanks hold material derived from children, pediatric biobanking is arguably the most efficient means for advancing critical clinical research given the limited availability of pediatric samples [2]. Only pediatric samples can assist researchers to understand and treat childhood diseases, human development, and the gene-environment interaction [2, 3]. Despite this, international regulation governing pediatric biobanking is generally inconsistent and unclear owing to the varying laws, policies, and guidance documents that regulate biological sample removal, biobank facilities, access to and privacy of genetic data, and the issues associated with lack of capacity to consent by most pediatric participants [4, 5]. There are estimated to be approximately 90 guidelines for biobanks published by national and international bodies generally [6]. However, few provide specific guidance on pediatric biobanking and these are not always binding and enforceable, nor are they consistent or comprehensive. There is a critical need for regulation and guidance on pediatric biobanking to be consolidated and clarified at an international level so that research—which will often be transnational—can more quickly, efficiently, and ethically be translated to useful information and treatment in pediatric care.

## Background 

Pediatric biobanking has nuanced ethical, social, and legal implications above those associated with biobanking generally. Many of these issues relate to the lack of capacity or vulnerability of child participants when their tissue enters a biobank, what role parents play in authorizing such participation, and how biobanks should deal with a child participant’s transition into adulthood.

For example, pediatric biobanking is complicated by a child’s generally assumed lack of legal capacity to consent to the collection and ongoing storage of their samples, particularly prior to the teenage years [7]. Furthermore, given the varying ages and maturity of children involved, questions arise as to whether, and to what extent, child participants *should* be involved in the original consent process and their assent or refusal respected [8–10]. Ethics committees may require both assent from the child after a certain age (e.g., 12 years and older) and consent from a legal guardian; however, this requirement can vary, and decisions still need to be made on the capacity of the child to assent. Given the ongoing nature of biobank storage, children also reach maturity and adulthood while the original research is ongoing or have reached maturity when new uses of the samples are proposed. Disagreement exists about gaining consent from a participant upon reaching adulthood due to the feasibility and costs of this process and whether human research ethics committees can waive this requirement [7, 11].

While it is accepted that parents (sometimes called “legal representatives/guardian” in documents) can consent on behalf of a child, the scope of this consent is contested [12]. Biobank samples are stored and can be used for extended periods of time, long after individual research projects end. Therefore, so long as a proposed project is approved by an ethics committee and the request respects the access governance requirements, samples can be used for new research projects, including ones that were unforeseeable at the time of consent [11, 13]. The quality of parental consent is also questionable. Research shows parents are often poorly informed about the biobanking process, including falsely believing in a therapeutic benefit to the child, not understanding that DNA can be obtained from biological samples, and falsely expecting the return of genetic information [14–17].

There are also pediatric specific concerns above the general privacy and confidentiality risks of biobanking. For example, while all biobanks are susceptible to data breaches, the limited availability of pediatric data and comparative lack of information about pediatric specific diseases create a critical need for translational and interdisciplinary sharing of biobank data [3, 10, 18]. Questions arise as to whether, and the extent to which, information derived about the child from the research should be shared with that child or their family. Arguments in favor reason that given the systematic lack of benefit for the participating child [19], particularly following the Declaration of Helsinki’s broadening of pediatric participation to include children who would not be directly benefited but from whom research would benefit other children of the same age or with the same condition [32], the child ought to reap any therapeutic or informational benefit from participating in a biobank [3]. Yet children are vulnerable as dependents and are therefore more susceptible to harms arising from genetic discrimination, such as familial or public stigma due to undesirable traits or unknown genetic relations and false beliefs about genetic determinism [11, 19, 20]. It is also arguable that children have a right to an “open future” and should have the opportunity to consent themselves to being given any information upon maturity [21]. This is further complicated given research outcomes have varying levels of validity and actionability, such as information relating to adult-onset diseases [3, 22].

These issues require specific and practical guidance to enable the safe, efficient, and ethical storage and sharing of pediatric samples and data. While regulation should be clear, comprehensive, and consistent, it can take a variety of forms with different levels of enforceability and influence over practice. Legal and non-legal measures can combine to regulate an area of practice and while both types can be influential, a hierarchy does exist which assumes specific laws hold greater influence compared with non-legal guidelines, mainly because stronger forms of compliance are available (e.g., breaches can result in offenses being committed). It should be noted that international conventions (sometimes referred to as “international law”) may be influential but are generally only enforceable if implemented in domestic law. Non-legal guidelines also sit in a regulatory hierarchy depending on the strength of recommendations made and the pressure to adopt them. While general laws exist in every country that can impact on the operation of pediatric biobanks, the lack of specificity of these laws and how they apply in the context of child participants mean that tailored regulation which forms a more specific framework to guide practice is necessary. For example, it is accepted that parents must consent on a child’s behalf; however, given the longitudinal nature of research and biobank storage, there may be uncertainty about the legal duration of that parental consent. With this in mind, this paper seeks to map the current state of international regulation relevant to pediatric biobanking to identify the extent to which it considers and consistently addresses these nuanced issues.

## Methods

Regulatory mapping was undertaken of international pediatric biobanking documents to find documents governing pediatric biobanks. Searches of Google Scholar was conducted to find international guidance documents on pediatric biobanking and children in clinical research or biobanking generally. Articles discussing the ethical, legal, and social issues of pediatric biobanking were also reviewed for references to regulatory documents until saturation. Located documents fell into four categories listed below from the most influential to the least influential.

- Enforceable: requires compliance enforced via domestic law, conditions of funding, or through compulsory membership
- Voluntarily binding: by requiring voluntary members to abide by guidelines
- Recommends: recommending specific guidelines for biobanks
- Information only: with bodies explicitly identifying relevant information or implicitly refraining from suggesting any particular solutions or advice

International guidance documents that specifically address biobanking are found in Table 1. International guidance documents that address pediatrics in research are in Table 2. National guidance documents addressing either pediatrics or biobanking are in Table 3.

**Table 1:** International guidelines on biobanking with specific mention or discussion of pediatrics

Document	Body	Year	Regulatory force	Discuss biobanking of genetic information	Parental consent	Child’s role in decision-making (consent/assent/dissent)	Need for reconsent on adulthood	Governance	Privacy
*Best Practices: Recommendations for Repositories Fourth Edition* [24]	International Society for Biological and Environmental Repositories	2018	Voluntarily binding	Some but not in relation to pediatrics (Guideline L2.6)	Required (Guideline L2.4.3.1)	Assent is required alongside parental permission (Guideline L2.4.3.1)	Biobanks can decide if consent is needed but it may be required in some circumstances (Guideline L2.4.3.2)	Human Research Ethics Committee should be consulted for guidance on whether re-consent is needed for matured minors or whether a waiver is appropriate (L2.4)	Considers privacy, but not to pediatrics specifically (L2.6)
*Declaration of Taipei on Ethical Considerations Regarding Health Databases and Biobanks* [30]	World Medical Association	2016	Recommends and sometimes enforceable^a^	Some but not in relation to pediatrics (Art 4)			Reasonable efforts should be made to attain consent (Article 13)	Research Ethics Committee may waive the need for consent in some circumstances (Article 16)	Considers privacy, but not pediatrics specifically (Article 11)
*International Charter of principles for sharing bio-specimens and data* [37]	Mascalzoni et al	2015	Recommends	Some but not in relation to pediatrics		No, only generally refers to legal frameworks in each country	Re-consent or notification with an opt-out clause is required	Ethics review boards can waive the need for reconsent where unfeasible or disproportionate to benefits	Considers privacy, but not pediatrics specifically
*Return of whole-genome sequencing results in paediatric research: a statement of the P3G international paediatrics platform* [43]	P3G International Pediatrics Platform	2014	Information only	Extensive discussion		The minor’s views should be sought and given age-appropriate consideration (Recommendation 2)		Oversight committee’s approach to governance is complicated by parental authority (page 4)	Information returns should be discussed in the consent process and only returned in specific conditions (Recommendations 1, 3, and 5)
*Developing a policy for paediatric biobanks: principles for good practice* [23]	European Society of Human Genetics	2013	Information only	Some limited statements (page 3)	Required, including discussion of relevance and scope (pages 3, 5)	Assent and dissent should be respected (page 6—Guideline 4)	Reconsent is required) (page 6—Guideline 5)	HREC involvement is required for the re-use of samples (page 5)	Can share information where reliable benefits to the child (page 6)
*Biobanks for Europe: A Challenge for Governance* [32]	European Commission	2012	Information only	Present but not about pediatrics	Permitted according to domestic laws (6.1.3.1)				
*Guidelines on Human Biobanks and Genetic Research Databases* [25]	Organisation for Economic Co-Operation and Development	2009	Recommends	Some but not in relation to pediatrics	Required according to law (Part II, 4. 26)	Assent may be permitted in some jurisdictions (Part II, 4.31)	Potentially required (Part II, 4.32)	REC may waive the need for consent (Part 22, 4.33)	Considers privacy, but not pediatrics specifically
*Recommendation of the Committee of Ministers to member states on research on biological materials of human origin (Rec(2006)4)* [42]	Council of Europe Committee of Ministers	2006	Recommends	Limited general statement (Article 17(1)(iii)			Minors can withdraw upon maturity (Article 15(3)		Confidentiality and the right to information are generally discussed but not specifically regarding minors (Art 25)
*International Declaration on Human Genetic Data* [33]	UNESCO	2003	Recommends	States that genetic data has a “special status” (Art 4)	Permitted according to domestic laws (Art 8(b))	The opinion of the minor should be considered in increasing amounts proportionate to their maturity and age (Art 8(c))			
*ISO 20387:2020* [46]	British Standard Institution/International Standardisation Organisation	2020	Voluntarily Binding	Some but not regarding pediatrics				Required (5.3)	

^a^For example, through official association with the World Health Organization and the United Nations, the WMA has some power where states require colleges and private associations to have compulsory membership. For example, all physicians in Albania are required to be members of the Order of Physicians of Albania, who are also members of the WMA

**Table 2:** International guidelines that are generally about clinical or medical research and discuss minors in relation to consent

Document	Body	Year	Regulatory force	Discuss genetic information/biobanking of GI	Parental consent	Child’s role in decision-making (consent/assent/dissent)	Need for reconsent on adulthood	Governance	Privacy/confidentiality
*International Ethical Guidelines for Health-related Research Involving Humans* [35]	Council for International Organizations of Medical Science & World Health Organization	2016	Recommends		Required (Guideline 17)	Age-appropriate assent must be sought, and deliberate objections respected, following tailored information (Guideline 17)	Required (Guideline 17)	REC can waive the need for parental permission (Guideline 17)	
*Convention for the protection of human rights and dignity of the human being with regard to the application of biology and medicine: Convention on Human Rights and Biomedicine* [36]	Council of Europe	1997	Voluntarily binding	Acknowledges genetic testing but not biobanking of genetic information	Required (Art 6(2)	Minors’ opinions should be considered according to their age and maturity, and any objection must be respected. The consent of the minor can be sufficient where sufficiently mature (Art 6, Explanatory Report paragraph 45)		Generally required (Art 16)	Generally considered but not specifically about pediatrics (Art 10)
*Convention on the Rights of the Child* [29]	United Nations	1989	Voluntarily binding		Required (Art 18)	When children are capable of forming their own views, they have the right to express their views in matters affecting them, and such views are given due weight in accordance with their age and maturity (Art 12(1))			Children shall not be subjected to arbitrary or unlawful interferences with their privacy (Art 16)
*Declaration of Helsinki: Ethical Principles for Medical Research Involving Human Subjects* [34]	World Medical Association	1964	Recommends		Required (Art 28)	Age-appropriate assent must be sought in addition to consent. Any dissent should be respected (Art 29)		Consent for collection, storage, or re-use can be waived by REC (Art 30 & 32)	Generally—all precautions must be taken (Art 24)
*Additional Protocol to the Convention on Human Rights and Biomedicine, concerning Genetic Testing for Health Purposes* [47]	Council of Europe	2008	Voluntarily binding	This is about genetic testing but not storage/biobanking	Yes (Art 12)	Where genetic testing is unnecessary, and a minor lacks consent, testing should be deferred until attainment of capacity (Art 1) The opinion of the minor must be considered in proportion to age and degree of maturity (Art 12(2))			Generally, but not specifically to pediatrics (Art 16)
*Working Document on Genetic Data* [48]	Council of Europe	2004	Voluntarily binding	This is about genetic information but not storage/biobanking	Yes—p. 12				
*Protection of Natural Persons with Regard to the Processing of Personal Data and on the Free Movement of Such Data and Repealing Directive 95/46/EC (General Data Protection Regulation)* [37]	European Parliament and the Council of the European Union	2016	Enforceable	Mentions genetic data (s 34)	Processing of the personal data of the child is lawful where consent is given by the holder of parental responsibility (Art 8(1))	Consent of parents is not necessary where the child has been offered preventative or counseling services (s 38)	Children should be able to withdraw their consent for processing of personal data even when they are no longer a child (s 65). This can be overridden in the public interest, to comply with the law, scientific, or historical research purposes, etc. (s 65)		Vaguely (s 39) but not in relation to pediatrics or biobanking

**Table 3:** National guidelines on biobanking with specific mention or discussion of pediatrics

Document	Body	Year	Regulatory force	Discuss biobanking of genetic information	Parental consent	Child’s role in decision-making (consent/assent/dissent)	Need for reconsent on adulthood	Governance	Privacy
*National Statement on Ethical Conduct in Human Research* [27]	National Health and Medical Research Council (Australia)	2023	Enforceable (independent statutory agency under the NHMRC Act (effective from 1 Jan 2024))	Yes (Chapter 3.3)	Yes (4.2.10)	Consent is required from young people in research who are sufficiently mature enough to understand (Chapter 4.2). Consent is required but not sufficient for young people with relative immaturity		HRECs must approve a plan to disclose or not disclose information about the health of donors, relatives, or the community (3.2.15) HREC can waive the need for consent in certain situations (3.3.14)	Generally—only return the information with analytic (scientific) and clinical validity, significance to the participant/relatives, and clinical utility (3.3.40) Privacy is generally discussed (3.3.58–61)
*Guidance Document for Human Research Biobanks and Associated Data* [45]	South Australia Health, Government of South Australia	2018	Recommends	Briefly, but not in relation to pediatrics				Certified HRECs can waive consent in many situations (Sect. 21)	The release of non-validated results is not recommended. If results are returned, they should be provided by a qualified genetic counselor (Sects. 25 & 26)
*MRC Ethics Guide: Medical research involving children* [41]	Medical Research Council (UK)	2014	Enforceable		Requires parental consent (5.1.2)	Where a child has sufficient understanding and intelligence (Gillick), they can consent (5.1.3). Any refusal must be respected		The Ethics Committee must consider clinical trials relating to pediatrics	Disclosure is only justified to prevent the actual or threatened suffering of the child. The consent form should request consent to inform and update GPs (5.2)
*Use and disclosure of genetic information to a patient’s genetic relatives under Sect. 95AA of the Privacy Act 1988 (Cth)* [40]	National Health and Medical Research Council (Australia)	2014	Enforceable	This is about genetic information but not storage/biobanking	Where a person is legally incapable of making decisions, follow domestic laws regarding legal representatives (p. 9)	Minors with sufficient understanding and maturity can give and withhold consent (in relation to using and disclosing genetic information) (p. 28)		Where parents refuse consent to disclose to genetic relatives, health practitioners may need to seek independent advice (p. 38)	Sharing of genetic information about a child depends on their age, maturity, emotional readiness, and mental capacity. At least one parent will likely be involved (p. 38)
*Pediatric Biobanks* [31]	Italian National Bioethics Committee	2014	Recommends	Thoroughly discussed	Partially restricted consent by a parental authority for specific research purposes is required (Art 2.1)	Age-appropriate information must be given to the minor and any dissent prevails over parental consent. More importance is placed on the minors as they mature (Art 2.1)	Minors upon maturity must be given the opportunity to withdraw or reconsent, modify, or renew their consent (Art 2.1)	The ethics committee must approve research generally (Art 2.1)	Third parties should be explicitly prohibited from accessing the data Information should only be returned in accordance with consent forms and under certain conditions (Art 2.1)
*Biobanks Information Paper* [38]	National Health and Medical Research Council (Australia)	2010	Information only	Yes, but not specifically to pediatrics	Yes (p. 33–4)	Researchers must respect the developing capacity of children and may find they have the necessary and sufficient capacity to consent (p. 33). Children without such capacity may “assent” to participation (p. 34). Dissent must be respected (p. 34)	Yes, further research should not continue when they reach maturity without their agreement (p. 34)	HREC approval is needed to waive the need for child assent or consent (p. 35)	Generally, but not specifically to pediatrics
*Guidelines for human biobanks, genetic research databases, and associated data* [46]	Western Australia Government	2010	Enforceable	Yes, these are guidelines for genetic research databases and biobanks	Yes, substitute consent can be obtained where the participant lacks capacity (Principle 4E)	Biobank policies must include whether and how the assent of a child should be taken and considered (Principle 4E: 4.14)	New consent can be obtained where substitute consent has previously been provided (but not specific to pediatrics) (4E)	Yes—generally about HRECS being involved as an oversight mechanism (7A)	Yes—generally (7B)
*Molecular Medicine Ireland Guidelines for Standardized Biobanking* [49]	Ireland and Northern Ireland	2010	Recommends	Briefly, but not in relation to pediatrics				All research must be approved by an HREC committee (p. 5)	Only clinically relevant information will be returned to the group of participants, not the individual (p. 5)
*Human biobanks for research* [50]	German Ethics Council	2010	Information only	Yes				Opinions of the ethics commission are necessary for using data from a biobank (p. 52)	Biobanks can report on individual research results to the participant (p. 39)
*Biobanks: obtainment, preservation, and utilization of human biological material* [39]	Swiss Academy of Medical Sciences	2007	Recommends	Acknowledges the storage of genetic data			It is necessary to ensure minors are able to exercise their rights as soon as they become capable of discernment (4.4)	Ethical committee plays a significant role in the regulation of research projects with human biological material	Donors have a right to be informed of diagnostically and therapeutically relevant results. Donors also have the right not to know (4.7)
*Guidelines for Genetic Biobanks* [44]	Italian Society of Human Genetics & Telethon Foundation	2004	Recommends	Yes, these are guidelines for genetic biobanks only	Consent must be provided by a legal guardian (3.1.2.2)	Opinion of minor should be taken into consideration according to age and maturity (Footnote 36)		Ethics Committee must provide consent for research (3.1.1)	The consent process should ask participants whether they wish to be informed of the results (3.1.1)
*Ethical guidelines for analytical research on the human genome* [26]	Ministry of Education, Culture, Sports, Science and Technology, the Ministry of Health, Labour and Welfare, and the Ministry of Economy, Trade and Industry (Japan)	2001	Enforceable	These are about research on genes, rather than biobanks	Consent can be obtained from minors aged 16 and over (but under 20 and unmarried) alongside parental consent. Consent must be obtained from the participant’s representative (s 8)	Researchers should give sufficient explanation in comprehensible terms for the minor’s understanding (s 8)		If there is fear of self-harm, discrimination, disownment, or impact on medical treatment, then the Ethics Review Committee’s opinion should be sought before disclosing information to the participant (s 9)	Minors over 16 may request and receive results where the chief researcher has given due consideration to the effect of the information on the minor. For minors under 16, the minor’s representative’s opinion should be sought first (s 9). The minor’s representative can request from the Ethics Review Committee to have the minor’s information disclosed to them (s 9)

## Results

While there are many international and national guidance documents on pediatric biobanking, Table 1, 2, and 3 show that most of these documents lack regulatory force, often fail to address specific issues of concern raised in the literature, and, when they do, can be inconsistent in the approach suggested. This can lead to confusion for researchers and those administering biobanks with pediatric material, as well as for participants and families.

### The nature of guidelines

Tables 1 and 3 show that the most specific pediatric biobanking guidelines are for information only or make recommendations that fall short of being voluntarily binding or enforceable. For example, the European Society of Human Genetics (ESHG) [23], International Society for Biological and Environmental Repositories (ISBER) [24], and Organisation for Economic Co-Operation and Development (OECD) [25] all have guidelines which are not binding. In contrast, the *Ethical guidelines for analytical research on the human genome* published by the Japanese Government are enforceable, but apply only nationally [26]. Some bodies have had their guidelines integrated into domestic regulation; for example, the Australian National Health and Medical Research Council (NHMRC) guidelines must be followed as a condition to accessing NHMRC funding [27]; ISBER’s guidelines are voluntarily binding on biobanks that seek accreditation using the American-based international biobank program “A2LA” which use the ISO 20387:2018 guidelines. The latter has been used to accredit biobanks in the USA, China, France, Italy, India, Poland, and South Africa [6]. The integration of these guidelines into regulation in a way that make compliance to some extent mandatory is, however, the exception rather than the norm.

Table 2 identifies the United Nations Convention on the Rights of the Child as part of the voluntarily binding backdrop in which biobanks conduct research, which pose several rights relevant to the conduct of pediatric research, the key one being the principle of a child’s “best interests” [28]. While all countries have signed and ratified, apart from the United States of America, enforceability only arises through ongoing monitoring and integration in domestic law. While some guidelines may be sufficiently recognized or respected either domestically or internationally to influence practice without such formal integration, this influence can be difficult to gauge.

### Absence of specific consideration of pediatric participants in biobanking guidance

Tables 1 and 3 show that there is insufficient pediatric specific guidance for biobanks. For example, ESGH and ISBER guidelines acknowledge that pediatric genetic data generally pose additional ethical concerns but do not expressly state the specific concerns nor provide ways in which to address those concerns [23, 24]. The OECD, ISBER, and World Medical Association’s (WMA) *Declaration of Taipei* guidelines simply note that biobanks can store genetic data and that biobanks should make efforts to protect the privacy of participant data [24, 25, 29]. While it is important to acknowledge that guidelines must be sufficiently broad so as to allow for flexibility and nuance of the specific contexts of biobanks and the research, apart from the ESGH guidelines, the common nuances of protecting pediatric privacy and confidentiality are also generally missing from those guidelines [23]. For example, while biobanks are generally required to protect the privacy and confidentiality of all participants equally, there is a lack of detail about whether it is permissible or desirable to return research information and results about the child to the parents who provided legal consent, rather than waiting for the child to mature and consent themselves, particularly children in middle adolescence who may be fully capable of making such decisions. This is also important given genetic data can reveal important information about familial relations or family members that may impact the child and their relatives if found out. There is also a lack of detail about whether and in what conditions pediatric information stored in biobanks can be shared with other biobanks or other researchers with only the Italian National Bioethics Committee (NBC) considering this and calling for a prohibition on third parties accessing pediatric biobank data [30].

Most biobank guidelines do acknowledge the need for someone to consent on behalf of the child [25, 31–35]. Some documents were broad, referring to a legal representative, but some were narrower, assuming parental authority was required. The scope of either authority was only defined by the NBC [30]; however, all others failed to identify whether parents could consent to all research, similar research, or just the research project or whether the child’s information could be shared to other biobanks and research teams.

Many documents failed to identify whether the child’s assent was required and whether consent was required upon reaching maturity. There was general acceptance that minors’ opinions should be increasingly considered as they mature, but no guidance as to whether this requires staying in contact with minors, especially given the one-off nature of biobank sample donations [23, 29, 30, 34, 36–39]. There was also little guidance on whether and what information should be relayed in the future to the child or their family in the consent process.

There was also a lack of explicit recommendations as to whether re-consent for subsequent research was required from the child (or who at the time of consequent research was now an adult) rather than the parent. For example, the WMA’s *Declaration of Taipei* requires consent for new research from persons who were not able to consent but have since gained capacity, but this is not raised in the context of minors [29]. Furthermore, the ESGH suggests that participants and their parents may be notified of new research, but only requires researchers to consult ethics committees before research changes “considerably” [23]. The Swiss Academy of Medical Sciences states that minors should be able to exercise their rights “as soon as they become capable of discernment” [39].

### Absence of specific guidance on pediatric biobanking in general pediatric research guidelines

Tables 2 and 3 show that there were many international research guidelines dealing with children that failed to provide guidance for pediatric biobanking. For example, the WMA’s *Declaration of Helsinki* [33] and the NHMRC documents identify the need for age-appropriate assent for children in clinical research but do not identify the biobank-specific need for consent when the minor matures due to the ongoing nature of biobank storage of samples [27, 38, 40]. While the Council for International Organizations of Medical Science & World Health Organization [34] guidelines on children in research require assent, respecting deliberate objections, and the need for re-consent generally in pediatric research, the nuances of biobanking are not identified, for example, the difficulty in re-consenting due to the nature of one-off donations, ongoing long-term research resulting in difficulties of re-contacting for consent, and the return of information unforeseeably known due to a lack of technology at the time of consent.

## Discussion: Divergence and contradictions

The most significant issue across these guidance documents was the conflicting standards and expectations in the following areas.

### Child and parental involvement

While many documents acknowledged the need for child involvement during the decision-making process, there was lack of consistency as to what “assent” meant, at what age assent must be sought, and whether assent was sufficient or just necessary in addition to parental consent. For example, the NBC guidelines only required biobanks to provide the minor with age-appropriate information, the United Nations’ Convention on the Rights of the Child only states that a child’s views must be given “due weight in accordance with their age and maturity” in matters that affect them [28], and the ESGH and ISBER guidelines required researchers to obtain assent from the child [23, 24, 30]. If biobanks were required to get consent from the child, there was conflict as to whether this must be a legal standard of capacity (i.e.. “*Gillick*” competency as required by the Medical Research Council (MRC) guidelines [41]) or because the child was “sufficiently mature” (i.e., required by the Council of Europe (1997) [35] and NHMRC guidelines [27]). The European Parliament and the Council of the European Union [37] state that parental consent is not necessary so long as the child has been offered preventative or counseling services in the context of processing personal data. Other guidelines only require dissent or “deliberate objections” to be respected rather than requiring formal assent [30, 34, 35] and many are silent on this issue altogether [29, 31, 36, 42].

Tables 1, 2, and 3 show that while minors should have the right to withdraw their consent upon maturity, there was divergence as to whether this was a requirement or an ideal. The NHMRC information paper [38] suggests that research should not continue without such consent whereas the ISBER guidance leaves this to biobanks to determine for themselves [24]. ISBER and OECD suggest consent “may” be required from mature minors but do not clarify the circumstances or criteria that may constitute conditions requiring consent [24, 25]. Many diverse exemptions are provided from the requirement to obtain consent. For example: the ESGH exempts gaining consent where it is unreasonable or disproportionate to the benefits [23]; the WMA’s *Declaration of Taipei* exempts gaining consent to protect the health of the population from identified, serious, and immediate threats [29]; and the European Parliament and Council of Europe permit exemption where it is against the public interest, to comply with law, or for scientific or historical research purposes [37]. The OECD simply notes that research ethics committees can waiver consent without identifying any reasons [25].

### Governance of biobanks

Guidance in relation to governance of biobanks is also varied.^1^ Some connote protective powers to ethics committees. For example, the ESGH requires the committee to review new research on the same sample or when the research changes significantly from the original description in the consent [23], and the NHMRC grants wide powers to ethics committees, such as to approve the plan for researchers to disclose or not disclose information in certain conditions to the donor, their relatives, or community [27]. However, the P3G international pediatrics platform guidance document notes that the capacity of clinical oversight committees to act as the gatekeepers on decisions to return information to patients is complicated by parental authority [43]. The WMA’s *Declaration of Helsinki*, although not specifically relating to pediatric biobanking, grants broad power to research ethics committees to waive the need for consent for collection, storage, or re-use generally.

### Return of information

Privacy and confidentiality issues in pediatric biobanking were inconsistent across the guidelines. Regarding the return of information to the child or their family, the ESGH, for example, states that the parents’ right to not know information is overridden by the child’s right to access any benefit from involvement in pediatric biobanking [23]. The MRC states that information should only be shared to prevent actual or threatened suffering to the child and, namely, to inform and update general practitioners [41]. The NBC, NHMRC, and P3G platform state that only information that is scientifically valid and reliable, clinically useful, and relating to childhood conditions should be returned [27, 30, 38, 43]. Japan’s guidelines state that information can be returned directly to minors over 16 so long as due consideration has been given to the effect of the information on the minor [26]. For minors under 16, their representative must be consulted regarding returning information to the minor, and the representative can also make a request for information disclosure from the ethics review committee.

Only three bodies consider a solution to this problem. The MRC sought to clarify this in consent forms to include circumstances of disclosure to pediatrics [41]. The Italian Society of Human Genetics & Telethon Foundation asks participants to identify in their consent forms whether they wish to be informed of results; however, these are not specific to pediatric participants [44]. The Government of South Australia requires all results pertaining to genetic information be returned through a genetic counselor, but is also not specific to pediatric participants [45].

## Conclusion

While there are some guidelines about pediatric biobanking, and others consider specific issues relating to pediatric research, the web of international documents relating to pediatric biobanking often conflicts between guidelines and is not comprehensive in its guidance. There is no universal consensus on the nuanced issues arising from pediatric biobanking, and given the translational nature of biobanking and their limited availability of samples, enforceable and cohesive regulation should be established to better facilitate this growing area of research. Better guidance is needed for researchers, biobank administrators, ethics committees, and child participants as they mature.

## Data Availability

No datasets were generated or analysed during the current study.

## Footnotes

1 Governance is defined differently in the guidance documents, for example, human research ethics committees, ethics committees, clinical ethics committees, and oversight committee. For the purposes of this paper, governance is broadly construed as an oversight body on research that ensures the research is conducted in accordance with ethics and law.
